# Semicircular Canal Influences on the Developmental Tuning of the Translational Vestibulo-Ocular Reflex

**DOI:** 10.3389/fneur.2018.00404

**Published:** 2018-06-05

**Authors:** Francisco Branoner, Hans Straka

**Affiliations:** Department Biology II, Ludwig-Maximilians-University Munich, Munich, Germany

**Keywords:** otolith organ, utricle, extraocular motoneuron, eye muscle, VOR, *Xenopus laevis*

## Abstract

Vestibulo-ocular reflexes (VORs) rely on neuronal computations that transform vestibular sensory signals into spatio-temporally appropriate extraocular motor commands. The motoneuronal discharge for contractions of the superior oblique eye muscle during linear translation derives from a utricular epithelial sector that is spatially aligned with the pulling direction of this muscle. In *Xenopus laevis*, the alignment is gradually achieved during larval development and requires motion-related semicircular canal afferent activity. Here, we studied the origin of semicircular canal and utricular signals responsible for the establishment and maturation of the extraocular motor response vector. Experiments were conducted on semi-intact preparations of *Xenopus* tadpoles before and after unilateral transection of the VIIIth nerve and in preparations of animals in which semicircular canal formation was prevented on one side by the injection of hyaluronidase into the otic capsule prior to the establishment of the tubular structures. Unilateral VIIIth nerve sections revealed that the excitation underlying the contraction of the superior oblique eye muscle during horizontal linear acceleration and clockwise/counter-clockwise roll motion derives exclusively from the utricle and the posterior semicircular canal on the ipsilateral side. In contrast, the developmental constriction of the otolith response vector depends on signals from the posterior semicircular canal on the contralateral side. These latter signals suppress directionally incorrect components that derive from the utricular sector perpendicular to the superior oblique eye muscle. This directional tuning complies with a stabilization of spatially correct utricular inputs that are aligned with the extraocular motor target muscle. In addition, misaligned signals are concurrently suppressed by semicircular canal-related commissural pathways from the contralateral side and through local interneuronal inhibitory circuits within the ipsilateral vestibular nuclei.

## Introduction

**Figure 1:**
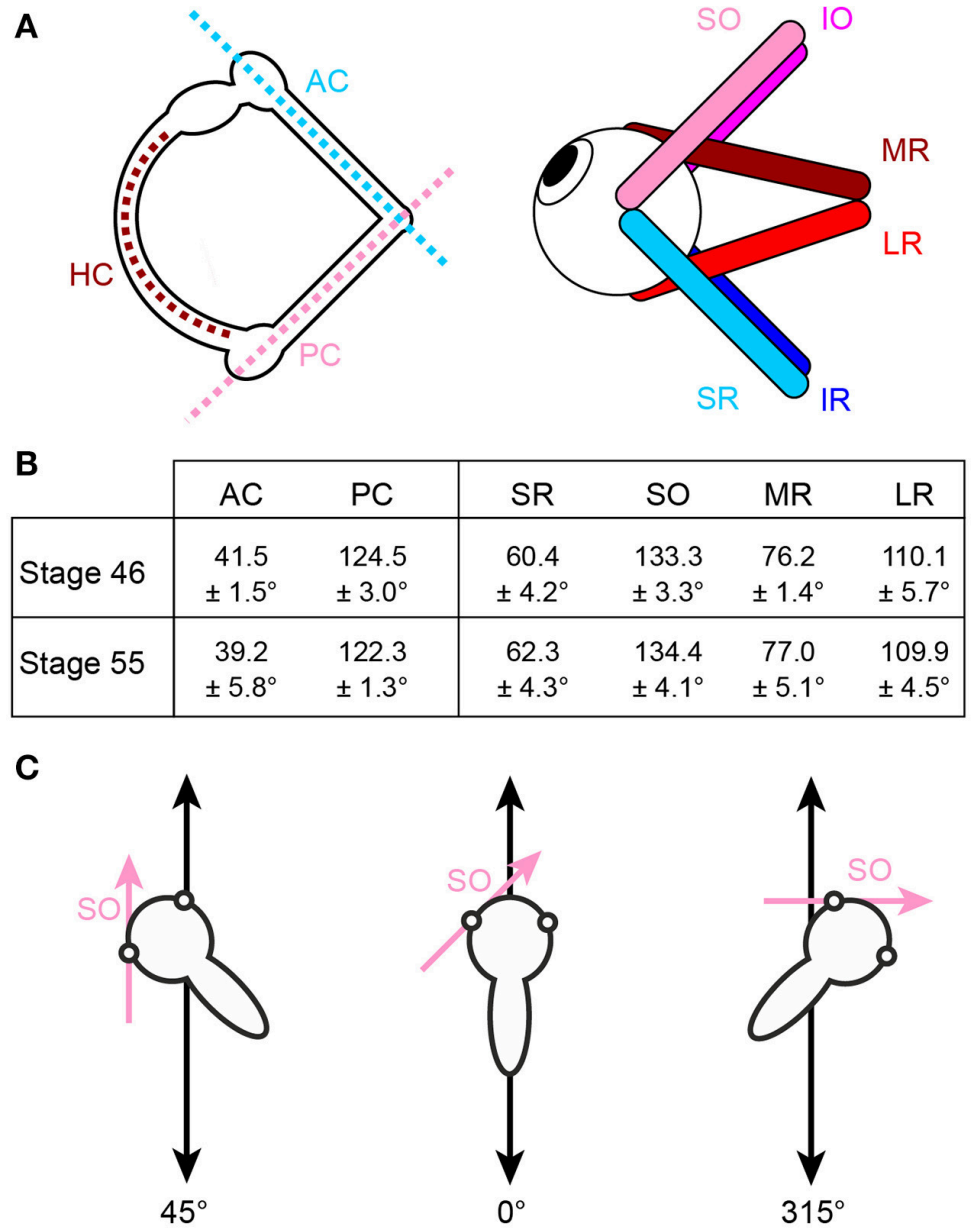
Arrangement of eye muscle pulling directions and semicircular canal planes. **(A)** Schematics depicting the spatial alignment of the three semicircular canals (left) and the six extraocular muscles (right) of the left inner ear and eye, respectively; note that color-coded antagonistic pairs of eye muscles are approximately aligned with individual semicircular canals. **(B)** Mean orientation of vertical semicircular canals and eye muscle pulling directions (± SEM) relative to the body length axis in tadpoles at developmental stage 46 and 55 (from *n* = 5 animals, respectively). **(C)** Exemplary orientations of semi-intact tadpole preparations either longitudinally on the linear sled (0°, middle) or in positions in which the SO eye muscle (magenta arrow) was either oriented parallel (left, 45°) or perpendicular (right, 315°) to the translational motion direction (double-headed black arrow). HC horizontal semicircular canal; AC, PC anterior, posterior vertical semicircular canal; IO, IR, inferior oblique, rectus muscle; LR, MR, lateral, medial rectus muscle; SO, SR, superior oblique, rectus muscle.

Gaze stabilizing eye movements derive mainly from the transformation of semicircular canal and otolith sensory signals into spatio-temporally adequate extraocular motor commands (1–5). Based on the motion detection mechanisms of the different types of inner ear organs, the performance of semicircular canal-derived reflexes is particularly robust at higher frequencies/accelerations while otolith-evoked reflexes are evoked during both high frequency motion stimuli as well as during slow changes of the head position within the gravitational field (6, 5). A major organizational principle of the vestibulo-ocular reflex (VOR) in all vertebrates is the approximate spatial alignment of semicircular canal orientations and eye muscle pulling directions that appears to be independent of the position of the eyes in the head [Figure 1A; (7–13)]. The respective signals from the different semicircular canals are relayed to the eye muscles via principal connections that are supplemented by auxiliary pathways to adjust for mismatches between semicircular canal planes and eye muscle pulling directions (9, 10).

At variance with the distinct directional sensitivity of each semicircular canal for angular acceleration, the utricle detects linear head acceleration in the horizontal plane over 360° and elicits a robust, three-dimensionally organized linear VOR that has been thoroughly studied over the past decades [see e.g., (6, 14–16)]. The omni-directional sensitivity for horizontal translation of this otolith organ as an entity, however, makes it difficult to conceive the mechanism by which hair cell epithelial sectors with particular directional specificity are connected during ontogeny to sets of VOR neurons with spatially matching extraocular motor targets. The orthogonal arrangement of semicircular canals [e.g., (17)], however, is well-suited as spatial reference frame for guiding a central convergence of directionally co-aligned angular and linear acceleration signals during embryonic and early post-embryonic development (18).

Natural head/body motion co-activates semicircular canal hair cells and those of directionally aligned utricular epithelial sectors [see Rohregger and Dieringer (13)]. The resultant afferent signals are combined and integrated within central vestibular circuits and distributed onto spatially matching sets of extraocular motoneurons in a pattern that is more or less independent of the position of the eyes in the head (7, 9, 10, 13, 19). The developmental establishment and tuning of these connections has been exemplarily demonstrated for motoneurons of the superior oblique (SO) eye muscle in *Xenopus laevis*, where the main excitatory drive originates from the posterior semicircular canal (PC) ipsilateral to the eye muscle and a spatially matching utricular epithelial sector. In this species, both sensory motion vectors are co-aligned with the pulling direction of the SO eye muscle (20). Directionally specific utricular responses in SO motoneurons in *Xenopus* tadpoles are only gradually achieved during the post-embryonic development by a tuning of initially omni-directional utricular responses in young larvae [stage 46; (20)]. Adequately tuned responses were first observed at stage 48–50, fully mature at stage 55 and coincide with the developmental onset and amelioration of semicircular canal function (20). This correspondence suggests that modulated semicircular canal afferent activity serves as a directional reference that allows utriculo-ocular connections to be spatially tuned. The employment of this reference frame potentially benefits from the relatively invariant spatial arrangement of semicircular canals and eye muscle pulling directions during larval development between stage 46 and 55 (Figure 1B). This scenario was confirmed by experiments in which the formation of bilateral semicircular canals was enzymatically prevented in *Xenopus* tadpoles (21, 20). This manipulation abolished modulated SO nerve activity during rotational motion, while utricle-dependent responses persisted, however, without directional preference even at late larval stages (20).

While the general impact of modulated angular acceleration signals on the tuning of the utricular vector of SO motoneuronal responses in *X. laevis* tadpoles was demonstrated previously, the origin of the semicircular canal signals and of the utricular signals with respect to the two inner ears is so far unknown. Our motivation therefore was to first reveal the side from which those utricular signals derive that activate SO motoneurons during horizontal linear acceleration. We then determined the side of the semicircular canal that causes the omni-directional utricular response vector to be directionally tuned during larval ontogeny and inferred from the vectorial restriction the specific identity of this semicircular canal. To identify the origin of the latter signals, we enzymatically prevented semicircular canal formation on one side in young larval *Xenopus*. Recordings of bilateral SO nerves at mid-larval stages demonstrated that angular acceleration signals contralateral to the SO eye muscle cause the utricular response tuning, suggesting that the inhibitory semicircular canal commissure causes an alignment of utricular signals with the SO eye muscle pulling direction.

## Materials and methods

### Animals

Experiments were performed on tadpoles (*n* = *35*) obtained from the in house breeding facility at the Biocenter-Martinsried of the Ludwig-Maximilians-University Munich. Animals were kept in fresh-water tanks at 17–18°C with a 12/12 h day-night cycle and fed daily with cyanobacteria (Bio-Spirulina Platensis, Naturwaren Blum, Germany). The age of tadpoles was determined according to the staging table of Niewkoop and Faber (22). Experiments were conducted on isolated *in vitro* preparations (23) and complied with the “Principles of animal care,” publication No. 86–23, revised 1985 of the National Institute of Health. Permission for the experiments was granted by the governmental institution at the Regierung von Oberbayern, Germany (55.2-1-54-2532.3-59-12; 55.2-1-54-2532-169-13).

### Enzymatic prevention of semicircular canal formation

The developmental formation of semicircular canals on one side was prevented by injections of the enzyme hyaluronidase into the otic capsule of post-embryonic larvae at stage 44 (prior to the normal formation of semicircular canals) as described previously (21, 20). Tadpoles were deeply anesthetized in 0.05% MS-222 (Pharmaq Ltd, UK) dissolved in tap water. Anesthetized animals were then mechanically secured to the Sylgard floor of a small Petri dish (5 cm diameter) at the level of the head and rostral tail with U-shaped insect pins and continuously supplied with oxygenated Ringer solution (75 mM NaCl, 25 mM NaHCO_3_, 2 mM CaCl_2_, 2 mM KCl, 0.5 mM MgCl_2_, and 11 mM glucose, pH 7.4). Injections were made with a micropipette (beveled tip 30°, 10–15 μm tip diameter, GB150F-8P, Science Products GmbH, Germany) filled with hyaluronidase (0.5 mg/ml; Sigma-Aldrich, France) dissolved in endolymph Ringer (24, 25). The micropipette was inserted into the center of the tadpole's right otic capsule with a piezo-driven triple-axis-micromanipulator (SMX-R-FS-50-HL, Sensapex Ltd., Finland) under visual control. This allowed pressure injection of the hyaluronidase solution (10 nl, 0.2 bar; *n* = 8) over a period of 10–15 s. After the injection, animals were kept for ~30 min in small tanks with oxygenated water for recovery from the anesthesia. Animals were subsequently transferred and individually housed in tanks (volume 500 ml) during further development until larval stage 55.

### Semi-intact preparations

Electrophysiological recordings of extraocular motor activity were performed *in vitro* on isolated preparations of stage 55 tadpoles. Animals were anesthetized as described above and decapitated at the level of the upper spinal cord. The lower jaw and the skin covering the head were removed and the cartilaginous skull opened from dorsal as described earlier (20). This procedure preserved all structural elements required for a functional VOR, including vestibular endorgans, central nervous pathways, and extraocular motor innervation of eye muscles. This allowed a natural activation of vestibulo-motor responses as under *in vivo* conditions (23). After isolation of the preparation, the trochlear nerve, which innervates the superior oblique (SO) eye muscle, was disconnected from its target muscle for extracellular SO nerve recordings. For the experiments, the preparation was fixed with insect pins to the Sylgard floor of a recording chamber (volume 5 ml) that was superfused with oxygenated Ringer solution (17 ± 0.1°C) at a rate of 1.3–2.1 ml/min. The chamber was tightly secured on a linear sled or a Hexapod (see below) that allowed applying sinusoidal linear and rotational acceleration stimuli in different directions relative to the head position (20).

### Electrophysiological recordings

Spontaneous and motion-evoked multi-unit activity was recorded from the disconnected SO nerve with individually adjusted glass suction electrodes (tip diameter ~40 μm; GB150F-8P, Science Products GmbH, Germany), fabricated with a horizontal electrode puller (P-87 Sutter Instruments Co., USA). The recorded signals were amplified (EXT 10-2F; npi electronics, Germany), digitized at 20 kHz (CED 1401, Cambridge Electronic Design Ltd., UK) and stored on a computer for later analysis.

### Sensory stimulation

Horizontal linear acceleration was applied by a custom-made linear sled [Tönnies, Switzerland; see Rohregger and Dieringer (13) for details]. The stimulus waveform was generated with spike2 software (version 6, Cambridge Electronic Design Ltd., UK) and consisted of sinusoidal horizontal linear acceleration at a frequency of 0.5 Hz and an amplitude excursion of ±5 cm (peak velocity: ±0.16 m/s; peak acceleration: ±0.49 m/s^2^) for 60 s (30 cycles). The stimulus was transferred to the linear sled by a data acquisition interface (micro3, Cambridge Electronic Devices Ltd., UK). The orientation of the recording chamber was changed systematically after each stimulus-trial along a given motion direction in steps of 15° to cover 360° in the horizontal plane (Figure 1C). This allowed the evaluation of the preferential spatial response vector of the multi-unit SO nerve discharge with respect to the utricular epithelial orientation. Angular motion stimuli were applied with a Hexapod (PI H-840, Physics Instruments; Karlsruhe, Germany) and consisted of sinusoidal rotations at a frequency of 1 Hz and peak velocities of ±60°/s (peak acceleration: ±400°/s^2^).

### Peripheral origin of utricular responses in SO motoneurons

The multi-unit discharge of the SO nerve on both sides was recorded before and after disconnection of the inner ear on one side. Accordingly, the right VIIIth cranial nerve was cut with a sharp lancet under visual control (*n* = 22) between the inner wall of the otic capsule and the entrance of the nerve root into the hindbrain without affecting brain structures. The preparation was allowed to recover for 30 min before testing the spatial vector orientation of SO nerve responses on both sides (ipsi- and contralateral to the transection).

### Data analysis

Electrophysiological recordings were analyzed with Spike2 Software (Cambridge Electronic Design Ltd., UK) using customized scripts in order to create peri-stimulus time histograms (PSTH) of the averaged extraocular motor nerve responses. The generated PSTHs served to determine the response magnitudes for each position of the preparation relative to the motion direction and allowed calculating the preferential orientation of the extraocular motor discharge during linear acceleration. As a measure for the directional preference, the response vector for each preparation was assessed by comparing the modulation depth during horizontal linear acceleration parallel to and perpendicular to the pulling direction of the SO eye muscle as described previously (20).

### Anatomy

At the end of the electrophysiological experiments, preparations (*n* = 8) were fixated for 4 h in a 0.1 M phosphate buffered (PB) 4% paraformaldehyde solution (Carl Roth, Germany) and subsequently transferred into 30% sucrose (Carl Roth, Germany; 0.1 M PB, pH 7.4) overnight. Horizontal sections through the entire head region at 50 μm thickness were made with a cryostat (Leica CM3050 S, Germany). Sections were mounted on slides and cover-slipped for bright-field microscopy. The imaging of inner ear structures in hyaluronidase-treated and unimpaired otic capsules as well as in the sham group verified the successful prevention of semicircular canal formation. The orientation of the semicircular canals and eye muscles was evaluated from photographed images of the transparent head from dorsal and indicated relative to the body length axis, respectively (Figure 1B).

### Statistics

Responses were averaged and are reported as mean ± standard error of the mean (SEM). Statistical differences between control/sham groups and animals that received unilateral hyaluronidase injections were calculated with the non-parametric Mann–Whitney *U*-test for unpaired parameters. Statistical differences of values from multiple groups were calculated with a non-parametric one-way ANOVA test (Kruskal–Wallis test). For comparison of differences in firing rate modulation during horizontal linear acceleration along different directions (parallel to or perpendicular to the SO eye muscle pulling direction) in a given group of animals, the non-parametric Wilcoxon signed-rank test for paired parameters was employed.

## Results

### Spontaneous and evoked neuronal discharge of SO motor units

**Figure 2:**
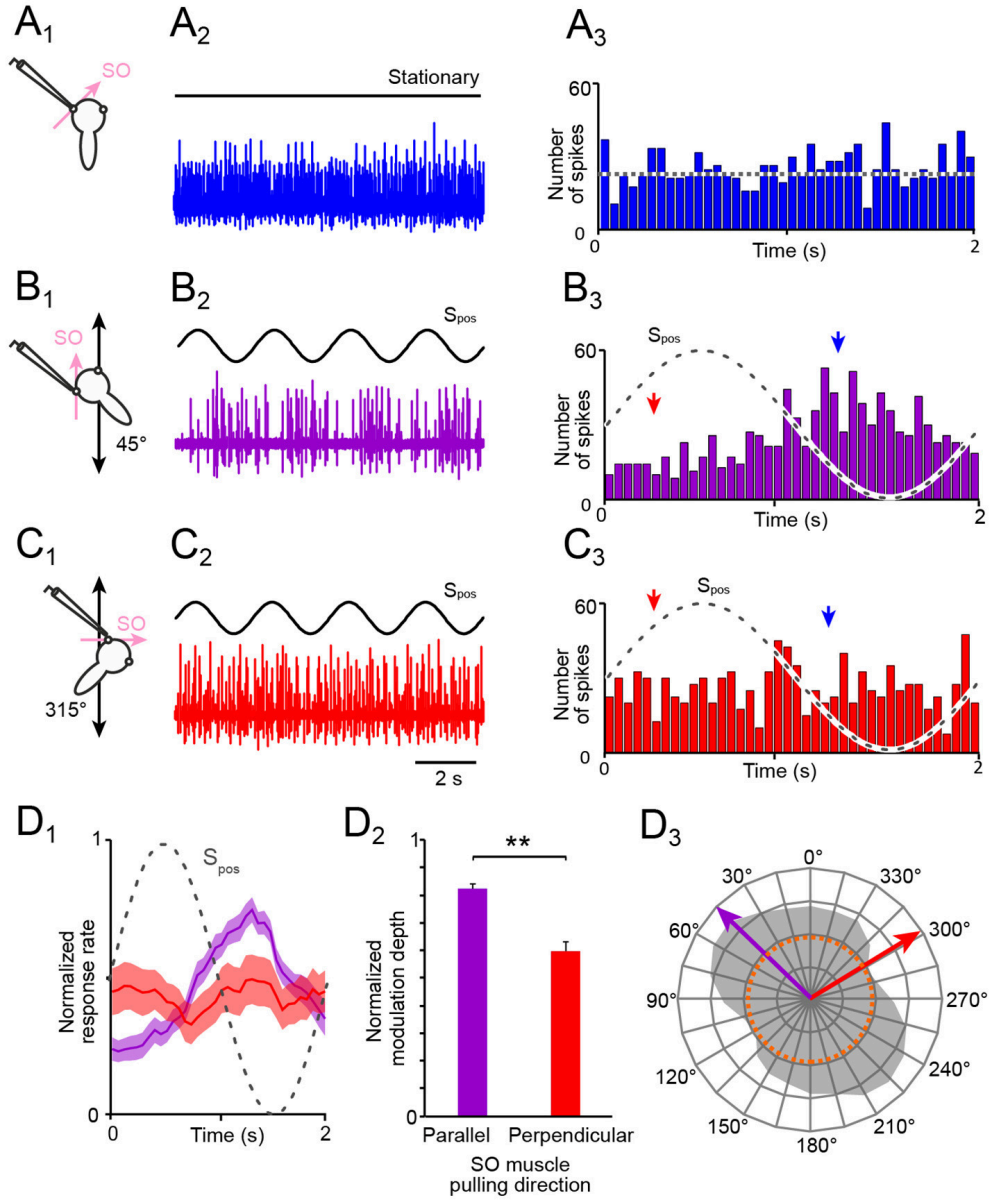
Spontaneous and motion-induced spike discharge of the SO nerve. **(A–C)** Multi-unit activity of the left SO nerve (color-coded traces in **A**_2_**,B**_2_**,C**_2_) in the absence of motion **(A**_1, 2_**)** and during four cycles of sinusoidal horizontal linear translation on a sled (S_pos_; black traces in **B,C**) at 0.5 Hz and 0.49 m/s^2^peak acceleration; preparations were positioned on the sled such that the SO muscle was oriented parallel **(B**_1_**)** or perpendicular **(C**_1_**)** to the translational motion direction; PSTHs (bin width: 50 ms) of spontaneous **(A**_3_**)** and averaged SO nerve firing rates over a single cycle (dashed sine waves) of sled motion (from *n* = 20–30 cycles), respectively **(B**_3_**,C**_3_**)**; the dotted line in **(A**_3_**)** indicates the average resting rate and the arrow heads in **(B**_3_**,C**_3_**)** the phase-time of the minimal (red) and maximal (blue) discharge. **(D)** Mean ± SEM of averaged SO nerve discharge modulation (**D**_1_; *n* = 8 preparations) over a single cycle (dashed sine wave) during linear motion parallel (violet) and perpendicular (red) to the SO muscle pulling direction; bar plot **(D**_2_**)** of normalized SO nerve firing rate modulation during translation parallel (violet) and perpendicular (red) to the SO eye muscle pulling direction (*n* = 8); significance of difference between the normalized response rates during motion in the two directions is indicated (^**^p ≤ 0.01; Wilcoxon signed-rank test); polar plot **(D**_3_**)** depicting the directional distribution of SO nerve discharge modulation magnitudes across 360° of translational motion (gray area) and preferential (violet arrow) and approximately orthogonal response vectors (red arrow); the dotted orange circle in **D**_3_ indicates the average resting rate. SO, superior oblique; S_pos_, stimulus position of the sled.

In stationary preparations, i.e., in the absence of imposed motion, the multi-unit activity of the SO nerve ranged in different preparations of stage 55 *X. laevis* tadpoles (*n* = *27*) from 7.7 to 37.2 spikes/s with a mean firing rate of 28.4 ± 10.2 spikes/s (Figures 2A_1−3_), compatible with multi-unit discharge rates of the SO nerve reported previously (20). During sinusoidal horizontal linear translation at 0.5 Hz and peak acceleration of ±0.49 m/s^2^ (Figures 1C, 2B_1−3_), the resting discharge became cyclically modulated as indicated by the typical multi-unit SO nerve recording (Figure 2B_2_). However, the magnitude of the peak firing rate depended on the particular orientation of the preparation relative to the acceleration direction (compare responses in Figures 2B_2_,C_2_). This dependency was quantified by calculating the modulation depth as the difference between maximal and minimal firing rates over a single stimulus cycle (red and blue arrow head in Figures 2B_3_,C_3_).

The modulation depth of the SO nerve discharge varied in different preparations (*n* = 27) from 18.0 to 63.3 spikes/s (mean ± SEM, 36.6 ± 14.8 spikes/s). This robust modulation was consistently evoked by sinusoidal linear translation along an axis that was parallel to the pulling direction of the SO muscle from which the recorded nerve was detached (45°; Figure 2B_1_). In contrast, the weakest SO nerve discharge modulation occurred during sinusoidal linear translation along an axis that was perpendicular to the pulling direction of this muscle (315°; Figure 2C_1_). The modulation depth (maximal—minimal firing rate), evoked by linear translation along this axis, varied in different preparations (*n* = *27*) from 12.0 to 24.5 spikes/s (mean ± SEM, 17.2 ± 8.9 spikes/s).

Comparison of the mean discharge modulation during horizontal linear translation along an axis parallel to (violet in Figures 2D_1, 2_) and perpendicular to the SO muscle pulling direction (red in Figures 2D_1, 2_) revealed a significant difference of the respective response magnitudes for the two directions (Figure 2D_2_; *p* < 0.01; Wilcoxon signed-rank test; *n* = 8). Systematic re-orientation of the preparations with respect to the direction of the linear translation in steps of 15° over 360° (gray area in Figure 2D_3_) yielded the directional preference (violet arrow in Figure 2D_3_) as well as the approximate direction of minimal modulation of utricle-evoked SO nerve spike activity (red arrow in Figure 2D_3_). The preferential response vector coincided with the pulling direction of the SO muscle as well as with the spatial orientation of the ipsilateral PC (20). This directional specificity served in the following as control for responses in preparations after an acute unilateral transection of the VIIIth nerve and in preparations obtained from unilateral semicircular canal-deficient animals.

### Transection of the VIIIth cranial nerve

**Figure 3:**
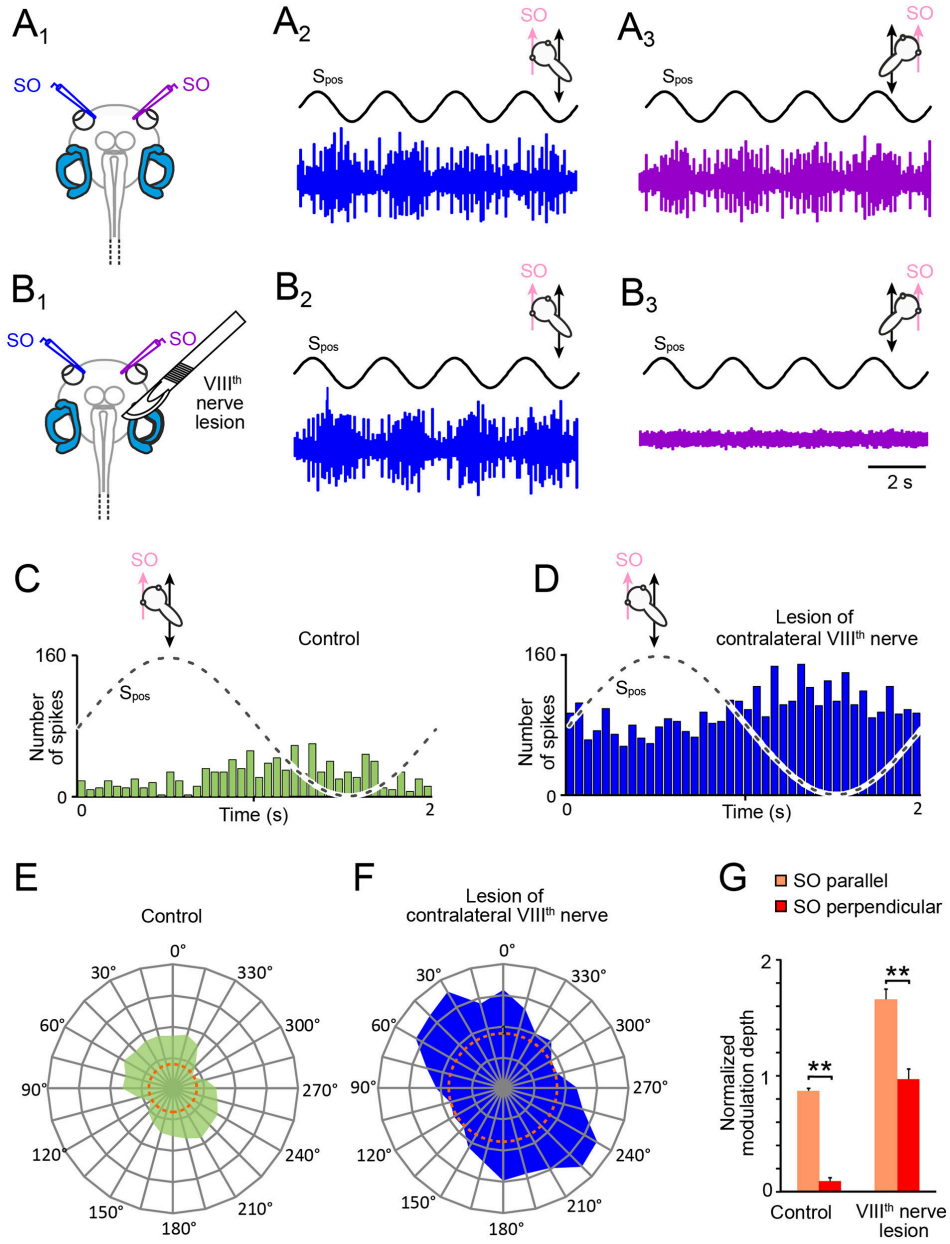
Consequences of unilateral VIIIth nerve lesions on motion-induced SO nerve firing rate modulation. **(A,B)** Discharge modulation of bilateral SO nerves during four cycles of sinusoidal horizontal linear translation on a sled at 0.5 Hz and 0.49 m/s^2^peak acceleration (S_pos_; black traces) before **(A**_1_**)** and after transection of the right VIIIth nerve **(B**_1_**)**; for maximal discharge modulation, preparations were positioned such that the left (**A**_2_**,B**_2_; blue traces) and right SO muscle (**A**_3_**,B**_3_; violet traces) were oriented parallel to the translation direction, respectively; note the complete loss of SO nerve spike activity on the ipsilesional **(B**_3_**)** and persistence of firing rate modulation on the contralesional side **(B**_2_**)**. **(C–F)** PSTHs (bin width: 50 ms) of averaged firing rate modulation over a single cycle (dashed sine waves in **C,D**) of sled motion (from *n* = 20–30 cycles) and polar plot of modulation magnitudes across 360° of translational motion **(E,F)** of the contralateral SO nerve before **(C,E)** and after VIIIth nerve transection **(D,F)**; data in **(C–F)** derive from the typical example depicted in **(A,B)**; dotted orange circles in **(E,F)** indicate the respective resting discharge rates. **(G)** Bar plot of normalized SO motor nerve firing rate modulation during translation parallel (orange) and perpendicular (red) to the SO eye muscle pulling direction (*n* = 8) before and after VIIIth nerve transection. SO, superior oblique; S_pos_, stimulus position of the sled.

Utricular signals that activate SO motoneurons during horizontal linear acceleration are aligned with the pulling direction of this eye muscle (20). However, given the 360° sensitivity of utricular hair cell epithelium for linear acceleration, these signals could theoretically derive from either one of the two utricles or from both sides. Therefore, in a first step we determined the origin of the utricle-derived discharge modulation of the SO nerve during sinusoidal horizontal linear acceleration (Figures 3A_1−3_) by a unilateral transection of the right VIIIth nerve in stage 55 tadpoles (Figures 3B_1−3_). This transection (right side in Figure 3B_1_) increased the resting discharge of the left, contralateral SO nerve instantaneously. Compared with the firing rate prior to the cut (31.2 ± 11.4 spikes/s; *n* = 22), the spontaneous discharge of ~55 spikes/s (55.4 ± 9.6 spikes/s) was significantly elevated (*p* < 0.01; Wilcoxon signed-rank test) corresponding to an increase by ~75%. In addition, the transection of the VIIIth nerve concurrently silenced the spontaneous discharge of the right, ipsilateral SO nerve, suggesting that the dominant tonic excitatory drive of SO nerve spike activity derives from the ipsilateral inner ear.

Compatible with the augmented resting discharge of the contralateral SO nerve, the spike discharge modulation during horizontal linear translation at 0.5 Hz and peak acceleration of ± 0.49 m/s^2^ also persisted after the VIIIth nerve cut (Figures 3B_2_,D). Moreover, the overall peak firing rate during translation parallel to the SO eye muscle (preferential direction) increased by ~90% (blue PSTH in Figure 3D) compared to controls (green PSTH in Figure 3C). This augmentation might be however simply a correlate of the elevated resting rate (compare orange dotted circles in Figures 3E,F), in particular since the discharge modulation depth before and after the lesion remained comparable (Figures 3C,D). In addition, the general difference in spike discharge modulation during translation parallel and perpendicular to the SO eye muscle pulling direction remained also relatively unaffected by the lesion (Figure 3G). Accordingly, the overall vectorial distribution and the preferential direction after the VIIIth nerve transection (Figure 3F) remained similar to the pre-lesion condition (Figure 3E), although the ratio of the modulation depths for the two orthogonal translation directions became less distinct after the lesion (Figure 3G).

In correspondence with the effect of the VIIIth nerve transection on the contralateral SO nerve, the spontaneous firing of the SO nerve ipsilateral to the lesion ceased completely after the cut and any discharge modulation during translational motion was abolished (Figure 3B_3_). The immediate and complete loss of spontaneous activity and motion-related responsiveness indicates that utricle-derived excitatory responses mediated by the SO nerve during horizontal linear acceleration predominantly if not exclusively originate from the ipsilateral inner ear. In contrast, the significantly increased resting rate and motion-evoked discharge modulation following removal of contralateral inner ear organs suggests that the latter exerts a tonic inhibitory influence on SO motoneuronal spike activity that becomes released after the lesion.

### Unilateral enzymatic prevention of semicircular canal formation

**Figure 4:**
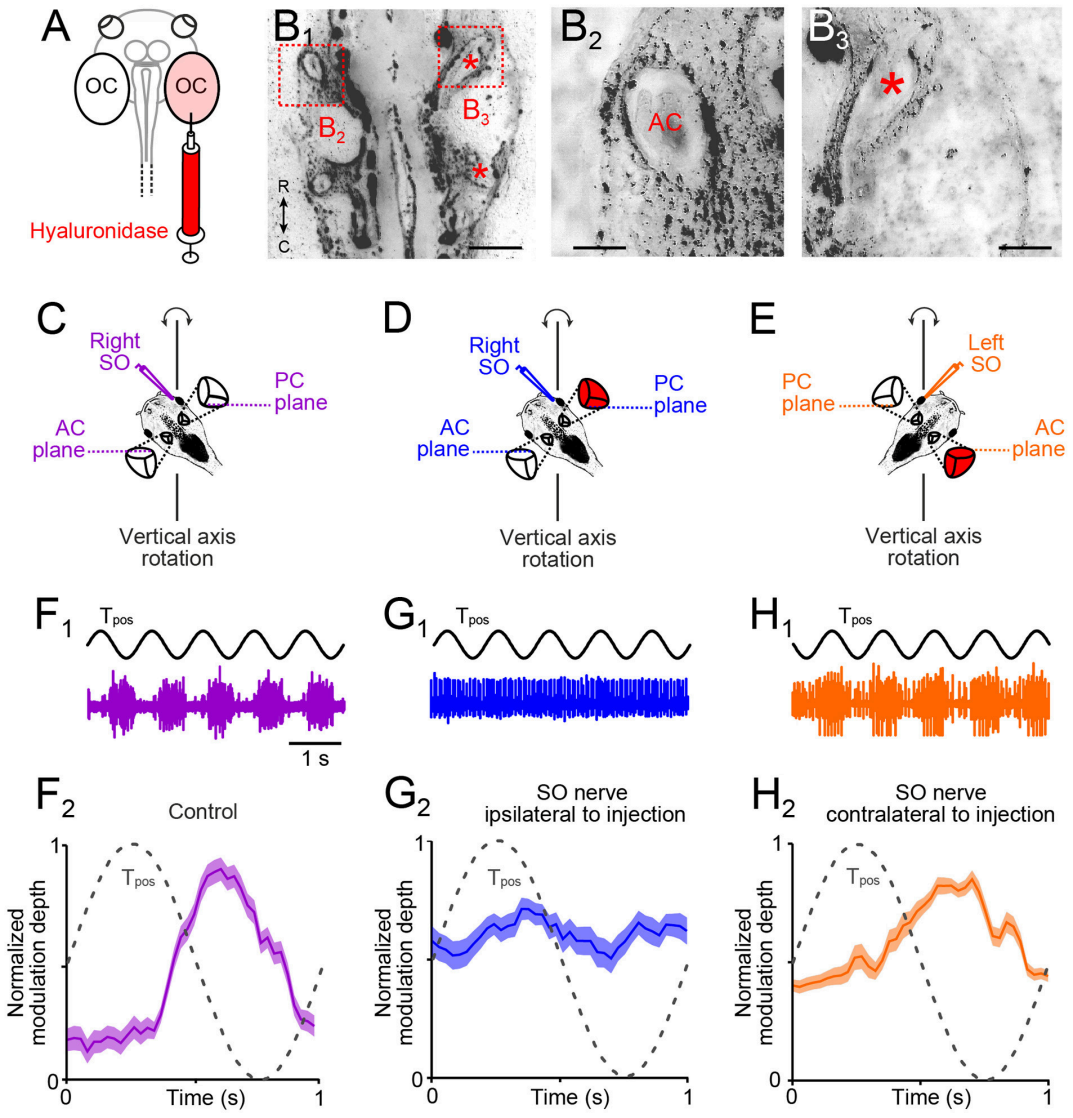
Morpho-physiological correlates of impaired angular VOR in unilateral semicircular canal-deficient *Xenopus* tadpoles. **(A)** Scheme depicting the injection of the hyaluronidase into the right otic capsule (OC, light red). **(B**_1_**–B**_3_**)** Overview **(B**_1_**)** of a horizontal section through the head of a stage 55 tadpole, which received a hyaluronidase injection into the right otic capsule at stage 44; higher-magnification of the boxed areas in **(B**_1_**)** of the left **(B**_2_**)** and right inner ear **(B**_3_**)**, indicating that semicircular canals developed on the left but not on the right side (red ^*^ in **B**_1, 3_ indicate putative locations of AC and PC). **(C–E)** Schematics of positional orientations of the preparations to record the right **(C,D)** and left **(E)** SO nerve during vertical-axis rotation; positional arrangements allow selective rotational stimulation at 1 Hz and ± 60°/s peak velocity of the right PC—left AC **(C,D)** or left PC—right AC **(E)**, without stimulation of the utricle. **(F)** Spike discharge modulation of the right SO nerve during five cycles of sinusoidal turntable rotation (T_pos_; black trace in **(F**_1_**)**) and averaged firing rate (mean ± SEM; **F**_2_) over a single cycle (dashed sine wave in **F**_2_) in a control. **(G,H)** Spike discharge modulation of the right **(G**_1_**)** and left SO nerve **(H**_1_**)** during sinusoidal rotation [T_pos_; black traces in **(G**_1_**,H**_1_**)**] in a preparation from a typical unilateral (right side) semicircular canal-deficient tadpole (red labeled inner ear); averaged firing rates (mean ± SEM; **G**_2_**,H**_2_**)** over a single cycle (dashed sine waves in **G**_2_**,H**_2_**)** show the loss of the excitatory **(G**_2_**)** or inhibitory response component **(H**_2_**)**; the averaged firing rate modulation over a single cycle in **(F**_2_**,G**_2_**,H**_2_**)** was obtained from 20–30 cycles, respectively. AC, PC, anterior, posterior vertical semicircular canal; SO, superior oblique; T_pos_, stimulus position of the turntable, C, caudal; R, rostral. Calibration bar represents 2 mm in **(B**_1_**)** and 0.5 mm in **(B**_2_**,B**_3_**)**.

The developmental alignment of the response vector between the activated utricular epithelial sector and SO eye muscle pulling direction in *X. laevis* tadpoles from an initially omni-directional response to directional adjusted responses depends on semicircular canal signals (20). These angular acceleration signals could derive either from the ipsi- or the contralateral inner ear. We therefore determined the role of ipsi- and/or contralateral semicircular canals to the developmental tuning of SO nerve responses during linear acceleration in animals that received an injection of hyaluronidase into one otic capsule at stage 44, prior to the normal morphological formation of these tubular structures (Figure 4A). As reported in an earlier study with bilateral injections (20), the enzyme prevented the establishment of all three semicircular canals in the injected inner ear (*n* = 8; Figures 4B_1, 3_). In contrast, semicircular canals developed normally within the non-injected otic capsule on the opposite side (Figures 4B_1, 2_) or after sham-injections of endolymph Ringer (*n* = 4). The absence of semicircular canals on the enzyme-injected side was verified in stage 55 tadpoles by recording the SO nerve discharge during rotational motion (frequency: 1 Hz; peak velocity: ±60°/s; Figures 4C–E). To exclude otolith-driven gravitoinertial contributions to the extraocular motor discharge modulation during rotation, preparations were spatially oriented such that either the right PC and left anterior semicircular canal (AC; scheme in Figures 4C,D) or the left PC and right AC (scheme in Figure 4E) were oriented horizontally. This orientation allowed selective stimulation of a given vertical semicircular canal pair during vertical-axis rotation without a concurrent modulation of utricular signals (20).

In such spatially oriented control preparations (scheme in Figure 4C), vertical-axis rotation (frequency: 1 Hz; peak velocity: ±60°/s) provoked a cyclically modulated discharge of the right SO nerve (violet trace in Figure 4F_1_). The robust spike discharge modulation (lower plot in Figure 4F_2_) complies with alternating excitatory and inhibitory influences of ipsilateral PC and contralateral AC signals on the SO nerve spike discharge (18). A comparable discharge pattern was obtained after sham-injections, compatible with the presence of normally formed semicircular canals (*n* = 4; not shown). However, animals with a unilateral hyaluronidase-induced semicircular canal deficiency exhibited a differentially impaired SO nerve discharge modulation (Figures 4D,E,G,H). Recordings of the right SO nerve (i.e., ipsilateral to the semicircular canal-deficient inner ear) during vertical-axis rotation (Figure 4D) caused a negligible modulation of the discharge (blue trace in Figure 4G_1_). In fact, the resting discharge appeared to be cyclically inhibited by signals from the left AC, i.e., on the intact side (Figure 4G_2_), while the normally prominent cyclic excitation was abolished. In a reciprocal manner, recordings of the left SO nerve (i.e., contralateral to the semicircular canal-deficient inner ear) during rotation (Figure 4E) provoked responses with a robust modulation of the peak discharge (orange trace in Figure 4H_1_). However, at variance with control recordings, cyclic inhibitory response components were largely absent (Figure 4H_2_). These results indicate, that, depending on the spatial orientation of the semicircular canal-deficient inner ear relative to the recorded SO nerve, either the rotation-evoked excitatory or inhibitory influence was abolished. This result is compatible with the spatially specific push-pull organization of the angular VOR in amphibians (20) as in other vertebrates (26). These experiments thus confirmed that unilateral hyaluronidase injection selectively prevents semicircular canal formation only on one side.

**Figure 5:**
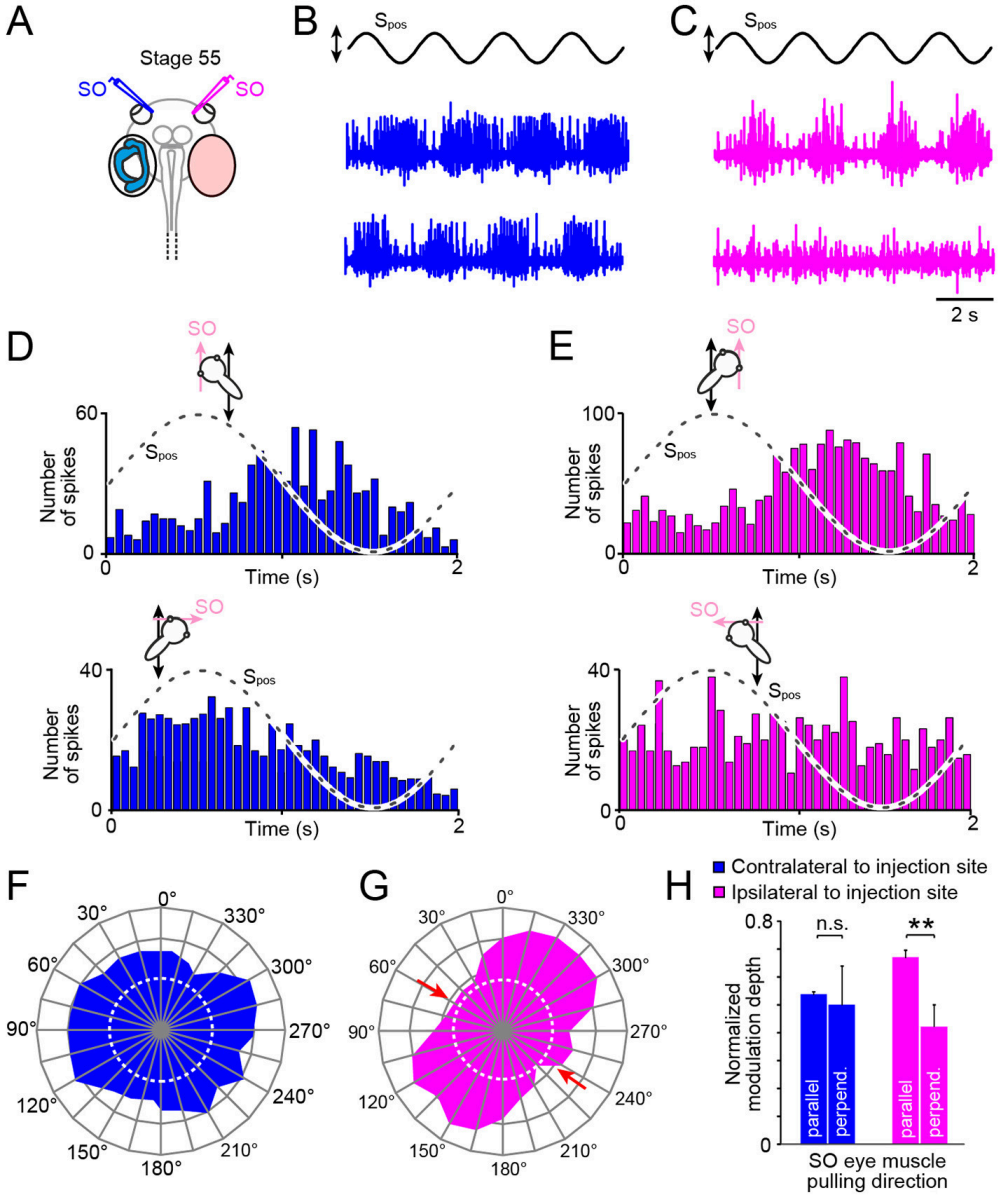
Impact of unilateral semicircular canal-deficiency on the developmental tuning of translational motion-evoked SO nerve responses. **(A)** Schematics depicting the presence of semicircular canals in the left and the absence thereof in the right inner ear in a stage 55 tadpole. **(B,C)** Spike discharge of the left **(B)** and right SO nerve **(C)** during four cycles of sinusoidal horizontal linear translation on a sled at 0.5 Hz and 0.49 m/s^2^peak acceleration (S_pos_; black trace) in a direction parallel (upper blue and magenta trace) and perpendicular to the SO eye muscle pulling direction (lower blue and magenta trace), respectively. **(D,E)** PSTH (bin width: 50 ms) of averaged firing rate modulation over a single cycle (dashed sine waves in **D,E**) of sled motion (from *n* = 20–30 cycles) of the left **(D)** and right SO nerve **(E)** during the two orthogonal translation directions. **(F–H)** polar plot of SO nerve discharge modulation magnitudes **(F,G)** and bar plot **(H)** of normalized firing rate modulation during translation along the two orthogonal directions of the left (blue) and right SO nerve (magenta); note the absence of directionally tuned responses of the SO nerve contralateral to the semicircular canal-deficient inner ear, indicated by the omni-directional vector plot (blue in **F**) and similar response magnitudes (blue bars in **H)** during translation parallel and perpendicular to the SO eye muscle pulling direction (*n* = 8), at variance with the corresponding responses of the SO nerve ipsilateral to the impaired inner ear (magenta vector plot in **(G)** and bar plot in **(H)**; ^**^p ≤ 0.01; Wilcoxon signed-rank test); red arrows in **(G)** indicate response components at spatial orientations, which became abolished during development. Dotted white circles in **(F)** and **(G)** indicate the SO nerve resting rates. n.s., not significant; SO, superior oblique; S_pos_, stimulus position of the sled.

We next studied the consequences of unilateral semicircular canal deficiency (Figure 5A) on the directional tuning of the utricular response vector of the SO nerve on both sides in these animals at stage 55 (Figures 5B–E). In stationary preparations, with both utricles oriented horizontally as in the control experiments, the spontaneous discharge of the left (mean ± SEM, 22.3 ± 12.1 spikes/s; *n* = 8) and right SO nerve (mean ± SEM, 24.8 ± 15.2 spikes/s; *n* = 8) were comparable (*n* = 8; *p* = 0.63; Wilcoxon signed-rank test) and similar to corresponding firing rates of unimpaired controls (*p* = 0.53; Mann–Whitney *U*-test) or sham-injected tadpoles (*p* = 0.58; Mann–Whitney *U*-test). Imposed sinusoidal horizontal linear acceleration (frequency: 0.5 Hz; peak acceleration: ± 0.49 m/s^2^) of the preparation caused in all semicircular canal-deficient animals a cyclic modulation of the SO nerve spike discharge on both sides as indicated by the typical recordings depicted in Figures 5B,C.

Systematic alteration of the horizontal position of the preparation in steps of 15° with respect to the direction of the horizontal linear acceleration revealed that the response of the SO nerve ipsilateral to the semicircular canal-deficient inner ear (right side; magenta trace and plot in Figures 5C,E) expressed a similar directional tuning as observed in age-matched controls (compare Figures 2D_3_, 5G). This was verified by quantifying the mean discharge modulation during horizontal linear translation along an axis parallel (mean ± SEM, 37.2 ± 12.6 spikes/s; *n* = 8) and perpendicular to the SO muscle pulling direction (mean ± SEM, 18.3 ± 10.5 spikes/s; *n* = 8). The statistical comparison yielded a significant difference in the respective response magnitudes during the two motion directions (*p* < 0.01; Wilcoxon signed-rank test; *n* = 8; magenta bars in Figure 5H). This indicates that the absence of semicircular canals ipsilateral to the SO eye muscle has no consequence for the developmental tuning of utricular responses in the SO nerve.

Accordingly, we next tested the spatial tuning of the SO nerve contralateral to the semicircular canal-deficient inner ear during horizontal translation (Figures 5B,D,F). The discharge modulation during sinusoidal horizontal linear acceleration (frequency: 0.5 Hz, peak acceleration: ±0.49 m/s^2^) of this SO nerve was robust and comparable to that on the other side, as indicated by the typical recordings from both sides of the same tadpole (Figures 5D,E). However, the depth of discharge modulation was largely independent from the position of the preparation relative to the direction of the translational motion (Figures 5D,F,H). The comparable magnitudes of the discharge modulation of the SO nerve irrespective of the orientation of the head indicated an omni-directional tuning of the utricular response vector (Figure 5F). This was confirmed by statistical comparison that yielded no significant difference in the magnitudes of the discharge modulation during translation parallel to (mean ± SEM, 31.3 ± 12.8 spikes/s; *n* = 8) and perpendicular to (mean ± SEM, 34.8 ± 13.4 spikes/s; *n* = 8) the SO muscle pulling direction (*p* = 0.74; Wilcoxon signed-rank test; *n* = 8, blue bars in Figure 5H). This tuning pattern is reminiscent of the condition observed in young tadpoles at stage 46 prior to the semicircular canal-dependent developmental tuning of utricular responses in the SO nerve (20). This strongly suggests that the physical integrity of semicircular canals on the contralateral side are responsible for the spatial tuning of the utricular response vector in SO motoneurons during development of *Xenopus* tadpoles.

## Discussion

Motor commands that evoke contractions of the SO muscle during translational motion derive from the transformation of ipsilateral utricular sensory signals. The response vector of the motoneuronal discharge is omni-directional in young tadpoles and becomes progressively aligned with the eye muscle pulling direction during larval development. This spatial tuning of motoneuronal activity requires modulated vestibular afferent discharge from semicircular canals of the contralateral inner ear.

### Semicircular canal and utricular signals for SO eye muscle contraction derive from the ipsilateral inner ear

**Figure 6:**
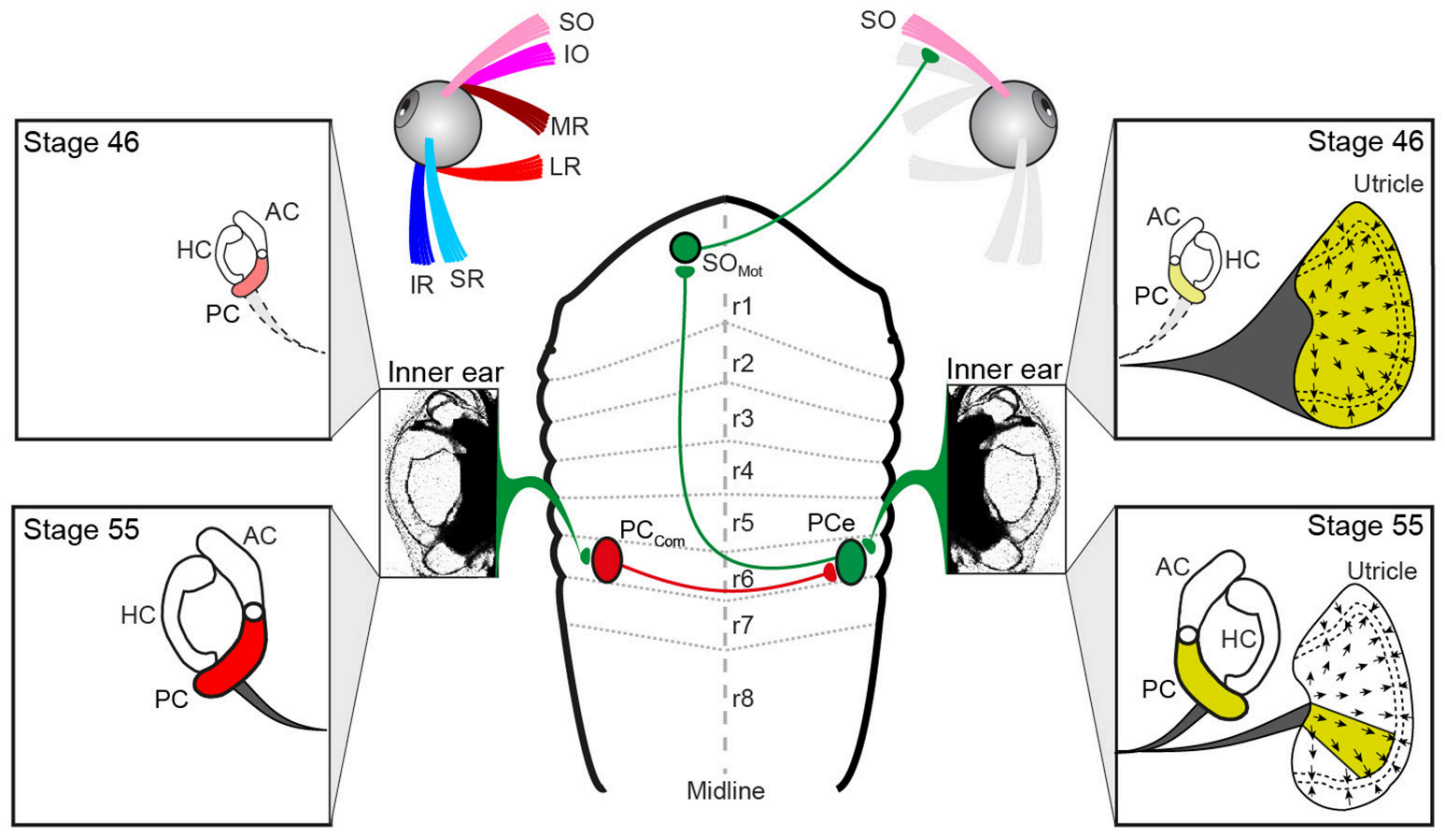
Schematics depicting the hindbrain connectivity of vestibular endorgans and the SO eye muscle along with the developmental changes in utricular and semicircular canal contributions during the developmental tuning of the SO nerve response vector. At stage 46, SO nerve responses derive from the entire utricle (yellow labeling on the right), whereas at stage 55, SO nerve responses derive from a utricular sector that aligns with the PC on the same side; the spatial restriction of the response vector in the direction perpendicular to the SO muscle derives from the contralateral PC (red labeling on the left) and is potentially mediated by midline crossing inhibitory pathways. AC, PC, HC, anterior, posterior vertical, horizontal semicircular canal; IO, inferior oblique; IR, inferior rectus; LR, lateral rectus; MR, medial rectus; PCe, excitatory PC neuron; r1-8, rhombomere 1-8; SO, superior oblique; SR, superior rectus, PC_com_, posterior canal commissural neuron; SO_Mot_, superior oblique motoneuron.

The developmental plasticity of gaze-stabilizing eye movements in larval *Xenopus* becomes only interpretable in the context of the basic, evolutionary conserved VOR blueprint (10, 27, 28). The essential goal of the VOR is the generation of eye movements by coordinated contractions of the six extraocular muscles of each eye to compensate for three-dimensional head motion perturbations and thereby to stabilize retinal image displacements. The sensory-motor transformation of vestibular signals underlying this reflex occurs for different spatial planes in distinct three-neuronal pathways between the inner ear and the eye muscles (see Figure 6 for excitatory three-neuronal PC-SO connectivity; (29)). The spatially specific synaptic connectivity between the sequential neuronal elements (vestibular afferents, second-order vestibular neurons, extraocular motoneurons) of this reflex pathway is the fundamental principle that guarantees the spatio-temporally precise functionality of VORs. An essential functional feature of the angular VOR specificity is the approximate matching spatial reference frame generated by the anatomical arrangement of the semicircular canals and the pulling directions of the eye muscles (7, 18). However, depending on the magnitude of misalignment between semicircular canal planes and eye muscle pulling directions, the principal connections underlying the angular VOR are supplemented by various auxiliary connections to adjust for species-specific spatial mismatches as elegantly demonstrated for the closely related water and grass frogs (30). During natural motion, semicircular canal stimulation usually occurs in combination with otolith organ activation. These signals converge in central VOR circuits to an extent that is surprisingly similar in species as distant as frogs (19) and primates (31). This combination of angular and linear acceleration signals particularly improves the performance of compensatory eye movements in the low frequency range (6, 32). The mutual assistance of the angular and linear VOR is facilitated by a spatio-temporally specific registration of the underlying semicircular canal and otolith signals that occurs at the level of the extraocular motoneurons and/or central vestibular neurons (11, 13).

The activation of such spatio-temporally correct compensatory eye movements during head/body rotation is based on VOR connections, at least for the angular VOR that comply with a so-called “push-pull” principle for antagonistic pairs of eye muscles (10, 26). The underlying neuronal pathways generally exert crossed excitatory and uncrossed inhibitory influences from a spatially aligned coplanar bilateral semicircular canal pair onto respective sets of extraocular motoneurons as shown for LR motoneurons in the abducens nucleus in species as different as frog (33) and cat (34). This connectivity also applies to SO motoneurons, which receive excitatory and inhibitory semicircular canal inputs from the spatially aligned contralateral PC and ipsilateral AC, respectively (10, 18, 35). However, because of the midline re-crossing trochlear nerve trajectory and the contralateral SO eye muscle innervation, the contraction occurs during rotation in the direction of the ipsilateral PC (excitation) and the relaxation during rotation in the direction of the contralateral AC (inhibition). Thus, with respect to the SO eye muscle, the excitatory semicircular canal drive originates from the PC of the ipsilateral inner ear (green pathway in Figure 6).

The vestibular sensory signals that provoke an increase of the discharge in the SO nerve during linear translation (e.g., Figure 2B_2_) could theoretically derive from the ipsi- or contralateral utricle. This is due to the fact that the hair cell epithelium of the utricle on each side is sensitive to motion across 360° (36). Transection of the VIIIth nerve ipsi- but not contralateral to the SO eye muscle, however, abolished the spontaneous firing as well as the translational motion-evoked spike discharge of the SO nerve (Figure 2B_3_). This indicates that the excitatory drive during linear translation derives exclusively from the ipsilateral utricle, as do PC signals during rotation (yellow labeling in Figure 6). If the matching, antagonistic epithelial sector of the contralateral utricle contributes an inhibitory component that facilitates SO eye muscle relaxation during linear motion in the opposite direction is possible, but yet to be experimentally verified. Thus, vestibular activation of the SO eye muscle during angular and linear translation of the head/body originates from the ipsilateral PC (10, 20) and a spatially matching epithelial sector of the utricle on the same side. The origin of these sensory signals from the same inner ear allows afferent inputs to be combined at the level of second-order vestibular neurons as indeed shown after electrical stimulation of individual inner ear nerve branches (19). This otolith-semicircular canal signal convergence in vestibulo-ocular projection neurons forms a convenient neuronal substrate at which the developmental tuning of the utricular response vector in SO motoneurons might be exerted.

### Semicircular canal signals responsible for tuning of utricular SO nerve responses derive from the contralateral inner ear

The developmental tuning of the utricular vector of SO motoneuronal responses between larval stage 46 and 55 was abolished in bilateral semicircular canal-deficient tadpoles (20). This demonstrates the necessity of the latter inner ear organs for the directional maturation of the translational VOR. Unilateral prevention of semicircular canal formation in the current study (Figures 4B_1−3_) further narrowed the origin of the latter signals. The utricle-derived response modulation of the SO nerve in stage 55 tadpoles remained omni-directional as those in stage 46 larvae (Figures 5D,F) in the absence of semicircular canals from the contralateral inner ear. In contrast, utricular responses of the SO nerve ipsilateral to the semicircular-deficient inner ear in the same animals became directionally tuned during larval development as those in controls (Figures 5E,G). This side-specific, differential effect on the spatial tuning of the SO nerve activity suggests an exclusive role of contralateral angular acceleration-related afferent signals in the developmental maturation. The unimpaired directional tuning of utricular SO nerve responses ipsilateral to the semicircular-deficient inner ear (Figures 2D_2_, 5H) in animals in which the tuning has already been established suggests an at most negligible contribution of semicircular canal signals on this side.

The developmental tuning of the initially omni-directional utricular responses establishes a matching directional specificity of utricular signals in the motoneurons with the pulling direction of the corresponding eye muscle. This sensory-motor alignment results from a removal of directionally incorrect utricular response components rather than from a further augmentation of spatially correct responses, i.e., parallel to the SO eye muscle (20). The semicircular canal-dependent reduction of those incorrect utricular response components during translational motion perpendicular to the SO eye muscle pulling direction must therefore derive from contralateral PC signals (Figure 6) as indicated by the impairment of the directional tuning after its ablation. Signals from this semicircular canal cause a “trimming” or “removal” of concurrently activated utriculo-ocular contributions during translation along this direction (Figure 6). The impact of contralateral PC signals on the sensory-motor transformation of spatially incorrect utricular signals could either be exerted directly at the level of SO motoneurons or more likely at the level of vestibulo-ocular projection neurons. In the latter case, the suppressive influence must involve spatially specific commissural connections (PC_com_ in Figure 6) that either directly or indirectly *via* additional local interneurons (37, 38) prevent vestibulo-ocular neurons with SO motoneuron targets from mediating signals of spatially incorrect utricular sectors.

### Potential cellular and circuit mechanism for the directional tuning

The directional tuning of utricular responses during *Xenopus* tadpole development might depend on a Hebbian-type plasticity “what fires together wires together” during co-activation of spatially aligned utricular and semicircular canal hair cells as previously suggested (20). Such a co-activation becomes gradually stronger during larval growth due to the concurrent increase in semicircular canal dimensions that facilitate the endolymph flow during rotational motion (25, 39). This in turn causes a more effective modulation of the afferent spike discharge. Such co-activation of afferent signals, however, appears not to cause a substantially larger excitation of SO motoneurons during translational motion alone (i.e., in the absence of semicircular canal activation) since peak response magnitudes remain comparable to those observed in young larvae (20). The tuning rather involves a gradual removal of spatially incorrect linear motion components (red arrows in Figure 5G) that derive from the orthogonal utricular epithelial sector (red labeling in Figure 6). Such an attenuation of particular utricular inputs, however, does not exclude a mechanism that also depends on Hebbian-type plasticity (40). In fact, a gradually stronger co-activation of signals from the orthogonal utricular sector and corresponding, spatially aligned PC might more efficiently activate inhibitory vestibular commissural pathways that then suppress or at least attenuate the incorrect utricular inputs in vestibulo-ocular projection neurons.

Vestibular commissural (41) along with local interneuronal inhibitory connections (42) offer convenient neuronal substrates for subtractive interactions with excitatory afferent inputs in central vestibular neurons (43). We therefore postulate a convergence of commissural inhibitory inputs that mediate signals from an “incorrect motion direction” during roll motion onto central vestibulo-ocular neurons that receive directionally corresponding ipsilateral excitatory utricular and semicircular canal afferent inputs. The concurrent co-activation of excitatory and inhibitory inputs would then gradually weaken the synaptic efficacy of those ipsilateral afferent response components that are not aligned with the SO eye muscle. During the trimming process, the respective synapses might be morphologically removed or transformed into silent but re-activatable synapses that persist into adulthood as suggested previously following partial VIIIth nerve lesions in adult frogs (44, 45). A potential mechanism to strengthen co-activated synaptic connections is the activation of NMDA receptors, which are abundant in second-order vestibular neurons (29) and known to facilitate longer-lasting synaptic plasticity. Accordingly, the reinforcement of spatially correct and elimination of spatially incorrect connections might follow similar rules as e.g., the maturation of visual circuits in the optic tectum of *Xenopus* tadpoles (46).

In conclusion, SO eye muscle contractions during roll motion derive from a transformation of sensory signals from the ipsilateral PC and a directionally co-aligned epithelial sector of the utricle on the same side. The spatial tuning of extraocular motor responses during development requires attenuating influences of the contralateral PC that cause a permanent removal of functionally inadequate utricular excitatory response components. This role of semicircular canals in the establishment of spatially specific connections between utricular hair cells and the SO eye muscle complies with the alignment of semicircular canal and extraocular motor reference frames that likely facilitate sensory-motor transformations underlying VORs.

## Author contributions

FB conducted the experiments, collected, and analyzed the data and made the figures. HS designed the experiments, outlined the figures, and wrote the manuscript.

### Conflict of interest statement

The authors declare that the research was conducted in the absence of any commercial or financial relationships that could be construed as a potential conflict of interest. The reviewer BY and handling Editor declared their shared affiliation.
